# Caspase recruitment domain-containing protein 9 (CARD9) knockout reduces regional ischemia/reperfusion injury through an attenuated inflammatory response

**DOI:** 10.1371/journal.pone.0199711

**Published:** 2018-06-25

**Authors:** Xing Qin, Matthew R. Peterson, Samantha E. Haller, Li Cao, D. Paul Thomas, Guanglong He

**Affiliations:** 1 School of Pharmacy, College of Health Sciences, University of Wyoming, Laramie, Wyoming, United States of America; 2 Department of Cardiology, Xijing Hospital, Fourth Military Medical University, Xi’an, Shaanxi, PR China; 3 College of Pharmaceutical Sciences, Soochow University, Soochow, Jiangsu, PR China; 4 Division of Kinesiology & Health, College of Health Sciences, University of Wyoming, Laramie, Wyoming, United States of America; Indiana University School of Medicine, UNITED STATES

## Abstract

Ischemic heart disease remains a leading cause of morbidity and mortality in the United States. Interventional reperfusion induces further damage to the ischemic myocardium through neutrophil infiltration and acute inflammation. As caspase recruitment domain-containing protein 9 (CARD9) plays a critical role in innate immune response and inflammation, we hypothesized that CARD9 knockout would provide protection against ischemia and reperfusion (I/R) injury through attenuation of acute inflammatory responses. C57BL/6 wild-type (WT) and CARD9^-/-^ mice were subjected to 45 min left anterior descending (LAD) coronary artery occlusion followed by 24-h reperfusion. Area at risk (AAR) and infarct size were measured by Evans blue and triphenyltetrazolium chloride (TTC) staining. Frozen heart sections were stained with anti-mouse GR-1 antibody to detect infiltrated neutrophils. Concentrations of cytokines/chemokines TNF-α, IL-6, CXCL-1 and MCP-1 were determined in heart tissue homogenate and serum by ELISA assay. Western immunoblotting analyses were performed to measure the phosphorylation of p38 MAPK. Our results indicate that following I/R, infarct size was significantly smaller in CARD9^-/-^ mice compared to WT. The number of infiltrated neutrophils was significantly lower in CARD9^-/-^ mice compared to WT. Levels of TNF-α, IL-6, CXCL-1 and MCP-1 were significantly reduced in heart tissue and serum from CARD9^-/-^ mice compared to WT. CARD9^-/-^ mice also exhibited significantly lower levels of phosphorylated p38 MAPK. Taken together, our results suggest that CARD9 knockout protects the heart from ischemia/reperfusion (I/R) injury, possibly through reduction of neutrophil infiltration and attenuation of CARD9-associated acute inflammatory signaling.

## Introduction

Despite continued progress elucidating underlying mechanisms in ischemic heart disease, it remains the leading cause of morbidity and mortality worldwide [1–4]. Following acute ischemic insult, damaged and dying cardiomyocytes expel a mélange of cytosolic materials including damage-associated molecular patterns (DAMPs) [5–7]. DAMPs are recognized by pattern recognition receptors (PRRs) of immune cells, activating a swift and severe local inflammatory response that induces further damage on the myocardial tissue at risk. Therefore, targeting the production of cytokines that propagate the inflammatory response and chemokines that attract infiltrating immune cells into the myocardium is emerging as a new frontier in the fight against I/R injury [8].

Neutrophils predominate the infiltrating immune cell population in the early inflammatory response following acute myocardial injury and provoke the greatest tissue damage [9, 10]. Necrotic cells in the damaged myocardium release a number of chemokines including C-X-C motif ligand 1 (CXCL-1) and monocyte chemoattractant protein 1 (MCP-1) which induce additional immune cell infiltration. The infiltrated neutrophils and afflicted myocardium secrete interleukin-6 (IL-6) and tumor necrosis factor-α (TNF-α), cytokines that potently drive inflammation and exacerbate myocardial injury [11–16]. However, even with an advanced understanding of the inflammatory signaling network, therapies targeting these inflammatory signals are still unable to prevent the injurious inflammatory response [7].

As a central regulatory protein in the innate immune response, caspase recruitment domain-containing protein 9 (CARD9) plays an indispensable role in the activation of transcriptional factors NFκB and p38 MAPK leading to induction of cytokines IL-6 and TNF-α [17–20]. CARD9 is preferentially expressed in immune cells such as neutrophils, but not cardiomyocytes, which may represent an advantageous approach for studying its signaling pathways without confounding issues of tissue specificity [18]. Innate immune responses are causally implicated in I/R injury [7, 8, 21]. We therefore postulated that CARD9 signaling may play a role in the acute phase of myocardial I/R damage.

With a homozygous CARD9 knockout mouse strain and an *in vivo* myocardial I/R model, the current study was undertaken to determine the role of CARD9 in the acute phase of myocardial I/R injury. A cytosolic scaffold protein, CARD9 activates transcriptional factors and induces cytokine production in immune cells. Therefore, findings from the current study may provide a potentially new therapeutic strategy to treat ischemic heart disease at a higher signaling control niche with respect to individual cytokines.

## Materials and methods

### Animals

C57BL/6 wild-type (WT) and CARD9^-/-^ mice were bred and housed in the animal care facility at the University of Wyoming College of Health Sciences. The animal protocol was approved by the Institutional Animal Care and Use Committee (IACUC) at the University of Wyoming. Procedures conformed to the federal guidelines for the humane and appropriate care of laboratory animals, Federal Law (89–544, 91–579) and all NIH regulations.

### Mouse heart ischemia and reperfusion (I/R) model

Mouse heart I/R surgery was performed as described previously [22–24]. Five months old WT and CARD9^-/-^ mice were anesthetized with an i.p. injection of ketamine (55 mg/kg) and xylazine (15 mg/kg), followed by tracheal intubation and ventilation with air at a tidal volume of 220 μL and respiratory rate of 120 breath/min. Left thoracotomy was performed and ischemia was induced for 45 min with a 7–0 silk suture passed around the left anterior descending (LAD) coronary artery and tied over a small piece of PE-50 polyethylene tubing. Reperfusion was subsequently achieved by loosening the suture and extracting the polyethylene tubing, while leaving the tie loose around the artery. Once the surgical intervention was complete, the chest was closed with a cruciate mattress suture pattern using 5–0 absorbable gut (Look 560) and the skin closed with a silk suture. Mice in the sham control group went through similar procedures without LAD occlusion. Twenty four hours after reperfusion, mice were re-anaesthetized and hearts were removed for infarct size measurement, as well as cytokine and protein analyses from heart tissue to determine the changes in inflammatory responses.

### Measurement of myocardial infarct size

The area at risk (AAR) and infarct size were determined by Evans blue and triphenyltetrazolium chloride (TTC) staining. The LAD was re-occluded and heart removed followed by injection of 1 mL of 5% Evans blue in phosphate buffered saline (PBS) solution through the aorta. Then the heart was frozen and cut into four slices with 2-mm thickness each using an acrylic mouse heart slicer matrix (Zivic LABS). The slices were stained in 1% TTC in PBS solution at 37 °C and fixed in 10% formaldehyde PBS solution for 24 h before being photographed. AAR and infarct area were contoured using the NIH ImageJ software (ImageJ; National Institutes of Health). AAR was expressed as percentage of the total ventricular area, and infarct size was expressed as percentage of AAR.

### Immunofluorescence staining of neutrophils in myocardium

To determine the number of neutrophils infiltrated, hearts were excised, embedded in OCT, frozen, and cryo-sectioned (7 μm thickness), and fixed at 4 °C for 5 min in acetone. Heart sections were stained with antibodies against granulocytes-1 (GR-1) (1:200, abD Serotec) for neutrophils and DAPI (0.1 μg/mL, Cell Signaling) for nuclei. Three images per heart were taken in the infarct area. Neutrophil numbers were quantified by dividing the number of GR-1 positive cells by the total number of cells (DAPI stained nuclei). Quantification was performed using ImageJ.

### Neutrophil isolation and co-culture with H9C2

To investigate the potential paracrine interaction between inflammatory cells and myocytes, neutrophils were isolated from WT and CARD9^-/-^ mice from a different cohort and co-cultured with myoblasts as previously described [25]. Briefly, Mice were injected i.p. with 2 mL 4% thioglycollate (TG) broth (Sigma Aldrich, St Louis, MO). Four hours later, mice were sacrificed by cervical dislocation and peritoneal cells were collected by lavage with 5 mL PBS. Red blood cells within the lavage were lysed with a lysis buffer containing 5 mL of 150 mM NH_4_Cl, 10 mM NaHCO_3_, and 1 mM Na_2_EDTA in dd-H_2_O. The isolated neutrophils were co-cultured with H9C2 cells in a transwell system (Corning^®^ Costar^®^ Transwell^®^ cell culture inserts No. 3412, VWR) as described previously [26–28]. Specifically, rat neonatal H9C2 myoblasts were seeded in the bottom compartment of the six-well transwell co-culture plate and neutrophils were seeded in the upper compartment of the transwell with a 0.4 μm porous membrane to allow for free exchange of soluble cytokines between the two chambers. Both cells were maintained in Dulbecco's modified Eagle medium with low glucose supplemented with 10% fetal bovine serum (FBS) and grown in a 37 °C incubator with 5% CO_2_. After 24-h co-culturing, the supernatant was collected. In a sub-group of co-cultured neutrophils, cells were treated with 10 μg/mL muramyl dipeptide (MDP) (A9519, Sigma Aldrich), a specific agonist of CARD9 in immune cells [18] for 24 hours, to study the role of CARD9 signaling.

### Western immunoblotting analyses

Proteins were extracted from the left ventricular risk area of WT and CARD9^-/-^ mice as well as H9C2 cells in RIPA (Millipore, Billerica MA) lysis buffer. Western immunoblotting analyses were performed as described previously [29]. Briefly, samples containing equal amounts of protein were separated on a 10% SDS-polyacrylamide gel. Proteins were transferred to nitrocellulose membranes and subsequently blocked with 5% BSA in Tris buffered saline (TBS)-tween 20. Membranes were incubated overnight at 4 °C with primary antibodies: phospho-p38 MAPK (1:1000, Cell Signaling), p38 MAPK (1:1000, Cell Signaling). Blots were washed with TBS-Tween-20 and incubated with horseradish peroxidase (HRP)-conjugated secondary antibody. Densities of protein bands were detected by chemiluminescence.

### Cytokine and chemokine measurement

Levels of IL-6, TNF-α, CXCL-1 and MCP-1 in cell culture supernatants, serum and heart tissue (10% wt/vol in ice cold PBS buffer) were determined using commercially available ELISA kits following manufacture’s specifications (IL-6, M6000B; TNF-α, MTA00B; CXCL-1, MKC00B; MCP-1, MJE00; R&D Systems).

## Statistical analyses

Data are presented as Mean ± SEM. Statistical significance was determined by 2-tailed Student's t-test in the case of two groups, and one way ANOVA followed by Newman-Keuls post-hoc comparisons when appropriate. A *p* value of less than 0.05 was considered significant for all the comparisons.

## Results

### CARD9 deficiency attenuated myocardial infarct size following I/R injury

**Fig 1:**
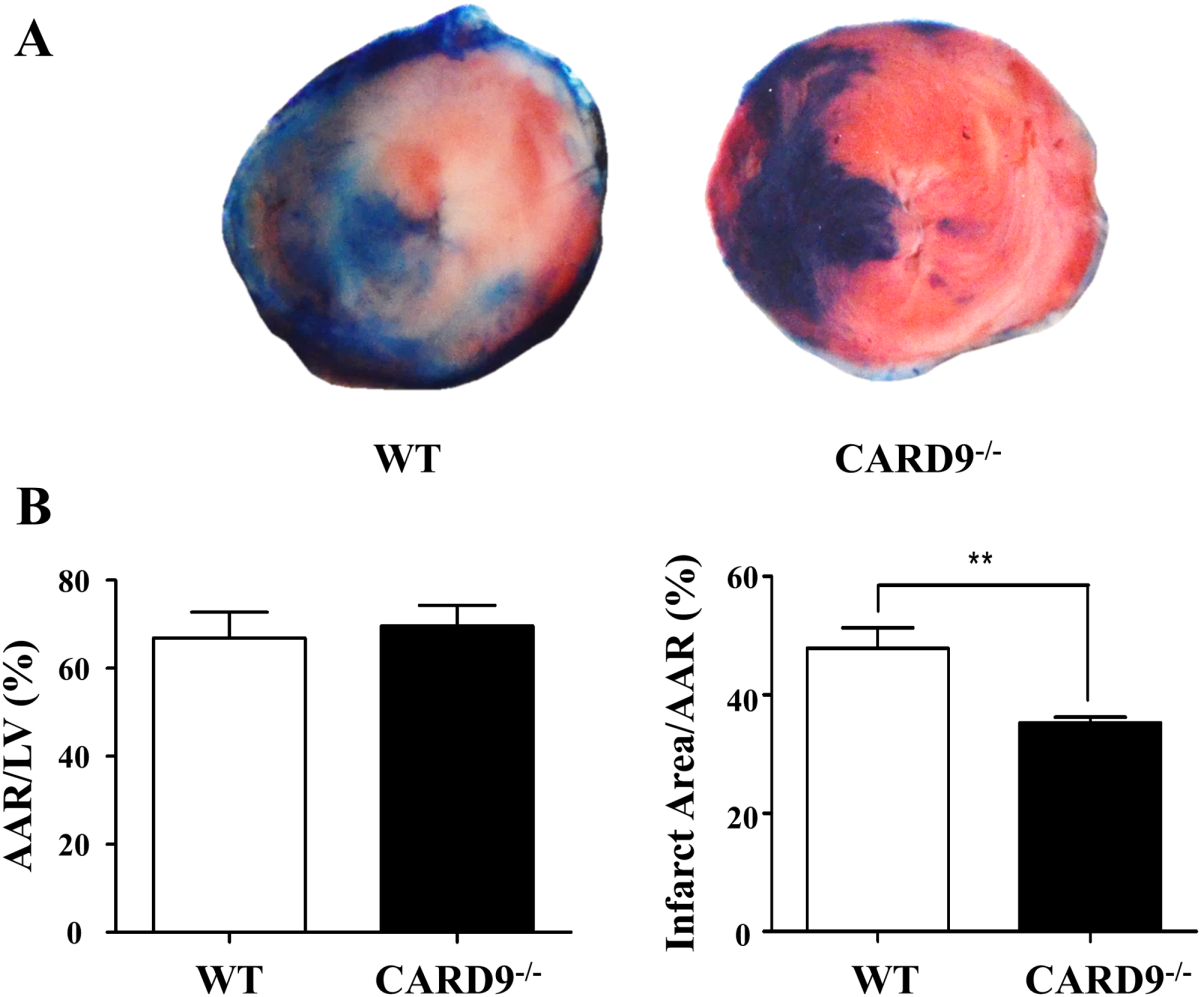
Measurement of myocardial ischemia and reperfusion (I/R) injury. C57BL/6 Wild-type (WT) and CARD9^-/-^ mice were subjected to 45 min occlusion of left anterior descending (LAD) coronary artery followed by 24 h of reperfusion. A: Representative heart sections stained with Evans blue and TTC to determine the total area of left ventricle (LV), area at risk (AAR, free of blue color) and infarct area (pale color); B: Analyses of the ratio of AAR/LV and infarct size (the ratio of infarct area/AAR) in the hearts of WT and CARD9^-/-^ mice. There was no significant difference in AAR/LV between the two groups, however the infarct size was significantly smaller in the CARD9^-/-^+I/R group than that in the WT+I/R group. Mean ± SEM, n = 5/group, **p < 0.01, CARD9^-/-^ vs. WT.

CARD9 is a central regulatory protein in the innate immune responses [18–20] as well as high fat diet-induced myocardial dysfunction [30]. We therefore hypothesized that CARD9 plays a detrimental role in the acute inflammatory responses associated with myocardial I/R injury. To test our hypothesis, I/R injury was determined in WT and CARD9^-/-^ mice as described in the Methods section. As shown in Fig 1, there were no significant differences in AAR/LV between the two groups indicating that the surgery generated similar AAR. However, infarct size was significantly smaller in the CARD9^-/-^+I/R than in the WT+I/R group demonstrating that CARD9 knockout protected the heart from I/R injury.

### CARD9 knockout attenuated neutrophil infiltration in heart tissue

**Fig 2:**
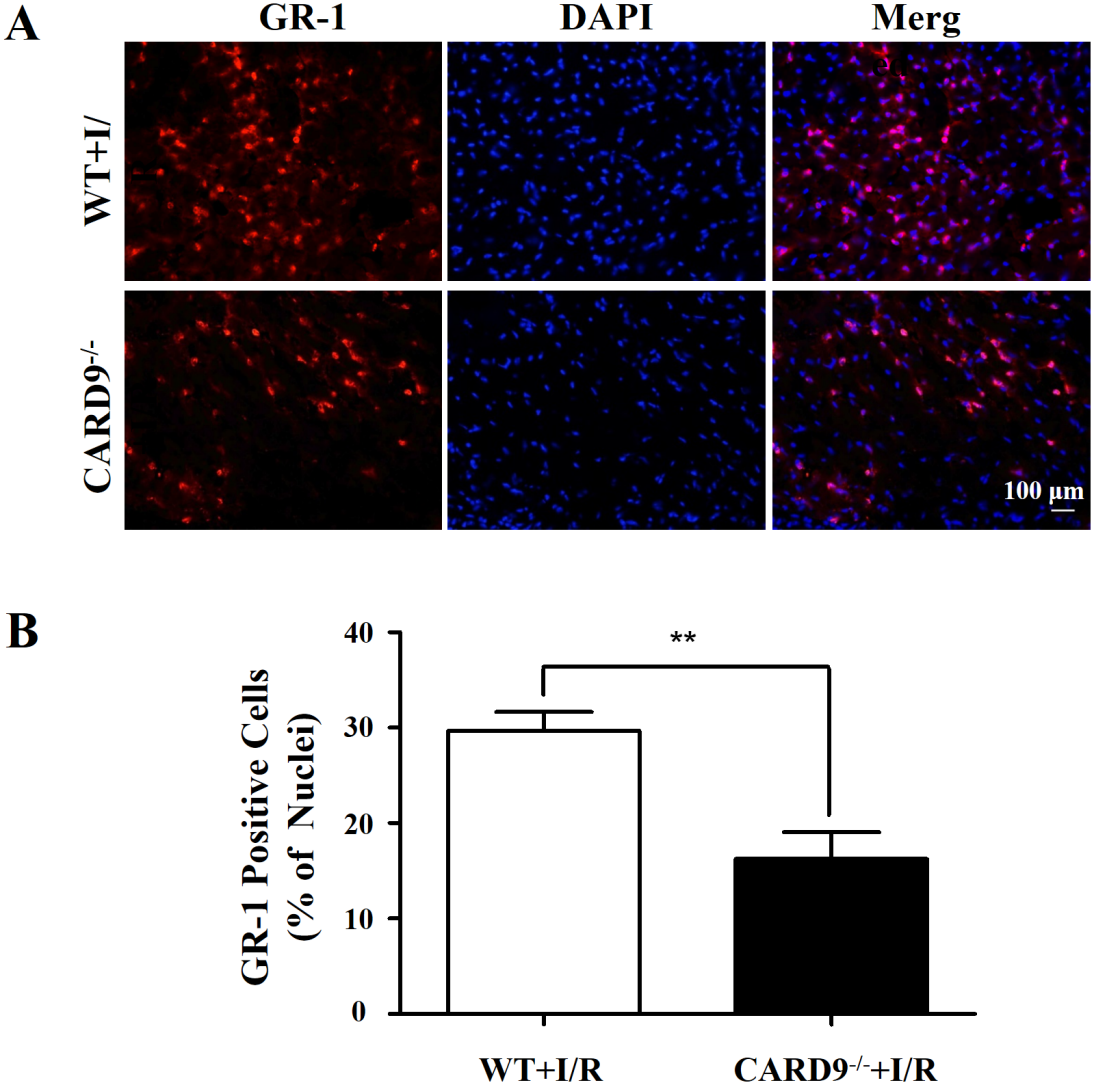
Immunofluorescence staining of neutrophils infiltrated in the heart tissue. Following 45 min LAD occlusion and 24-h reperfusion, hearts from WT and CARD9^-/-^ mice were cryo-sectioned (7 μm) and stained with antibodies against GR-1 for neutrophils (red) and DAPI for nuclei (blue). A, Representative heart sections showing GR-1, DAPI and merged staining images of neutrophils and nuclei; B, Analyses of the number of neutrophils as percentage of the total cell nuclei in the section. The number of infiltrated neutrophils in the WT mouse heart was significantly higher than that in the CARD9^-/-^ mouse heart. Mean ± SEM, n = 3/group, **p < 0.01 vs. WT.

Neutrophil infiltration following acute inflammation contributes to tissue injury [13, 31, 32]. To determine the potential mechanisms underlying the protective effect of CARD9 knockout against myocardial I/R injury, immunofluorescence staining was performed to measure the number of neutrophils infiltrating the myocardium. As shown by the red staining in Fig 2, GR-1 positive neutrophils infiltrated the infarcted area of left ventricle. CARD9 knockout significantly reduced the infiltrated GR-1 positive cell number suggesting a potential mechanism for CARD9 knockout-afforded myocardial protection.

### CARD9 deficiency attenuated chemokine production

**Fig 3:**
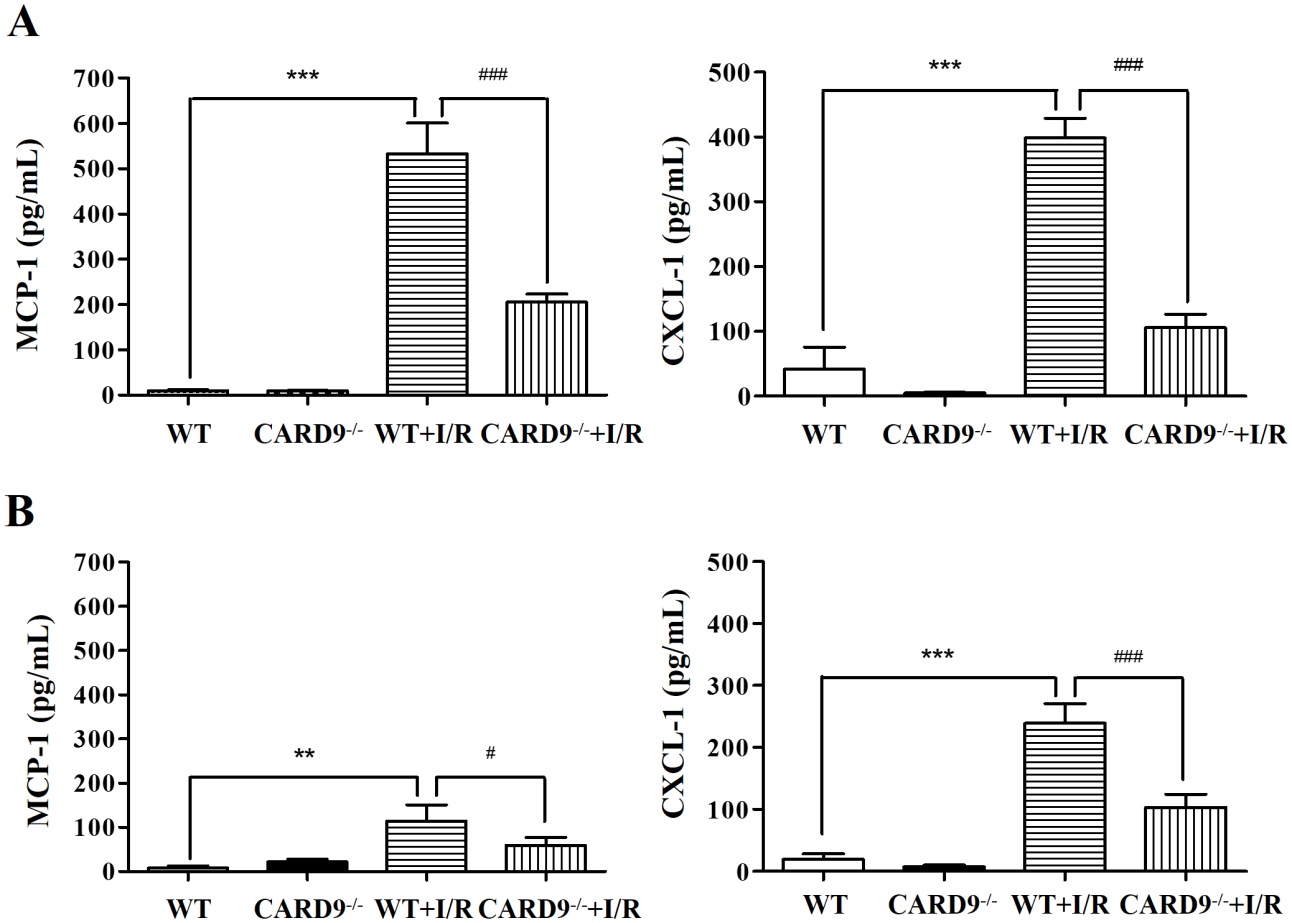
Measurement of chemokine production in the heart and serum following I/R injury. After 24 h reperfusion, heart tissue and blood were harvested from the WT and CARD9^-/-^ mice. Monocyte chemotactic protein 1 (MCP-1) and chemokine C-X-C motif ligand 1 (CXCL-1) in heart tissue homogenate and serum were measured using commercial ELISA kits. A, Concentrations of MCP-1 and CXCl-1 in the heart tissue; B, concentrations of MCP-1 and CXCL-1 in serum. CARD9 deficiency significantly reduced concentrations of chemokines MCP-1 and CXCL-1 in the heart tissue and serum following I/R injury. Mean ± SEM, n = 4 /group, **p < 0.01, ***p < 0.001 vs. non-I/R; ^#^*p* < 0.05, ^##^*p* < 0.01, ^###^*p* < 0.001 vs. WT.

As CARD9 deficiency attenuated neutrophil infiltration into the heart, we examined if neutrophil-associated chemokine production was suppressed in the CARD9^-/-^ mouse hearts following I/R injury. As shown in Fig 3, I/R injury induced significant increases of MCP-1 and CXCL-1 in heart tissue and serum compared to the sham control group. Interestingly, the increases in MCP-1 and CXCL-1 were significantly attenuated in CARD9^-/-^ mice implying a role for this protein in the release of these chemokines. It is pertinent to note that the levels of MCP-1 and CXCL-1 in heart tissue were much higher than that in serum suggesting a potential paracrine contribution from neutrophils infiltrated into the heart.

### CARD9 deficiency attenuated I/R-induced phosphorylation of p38 MAPK

**Fig 4:**
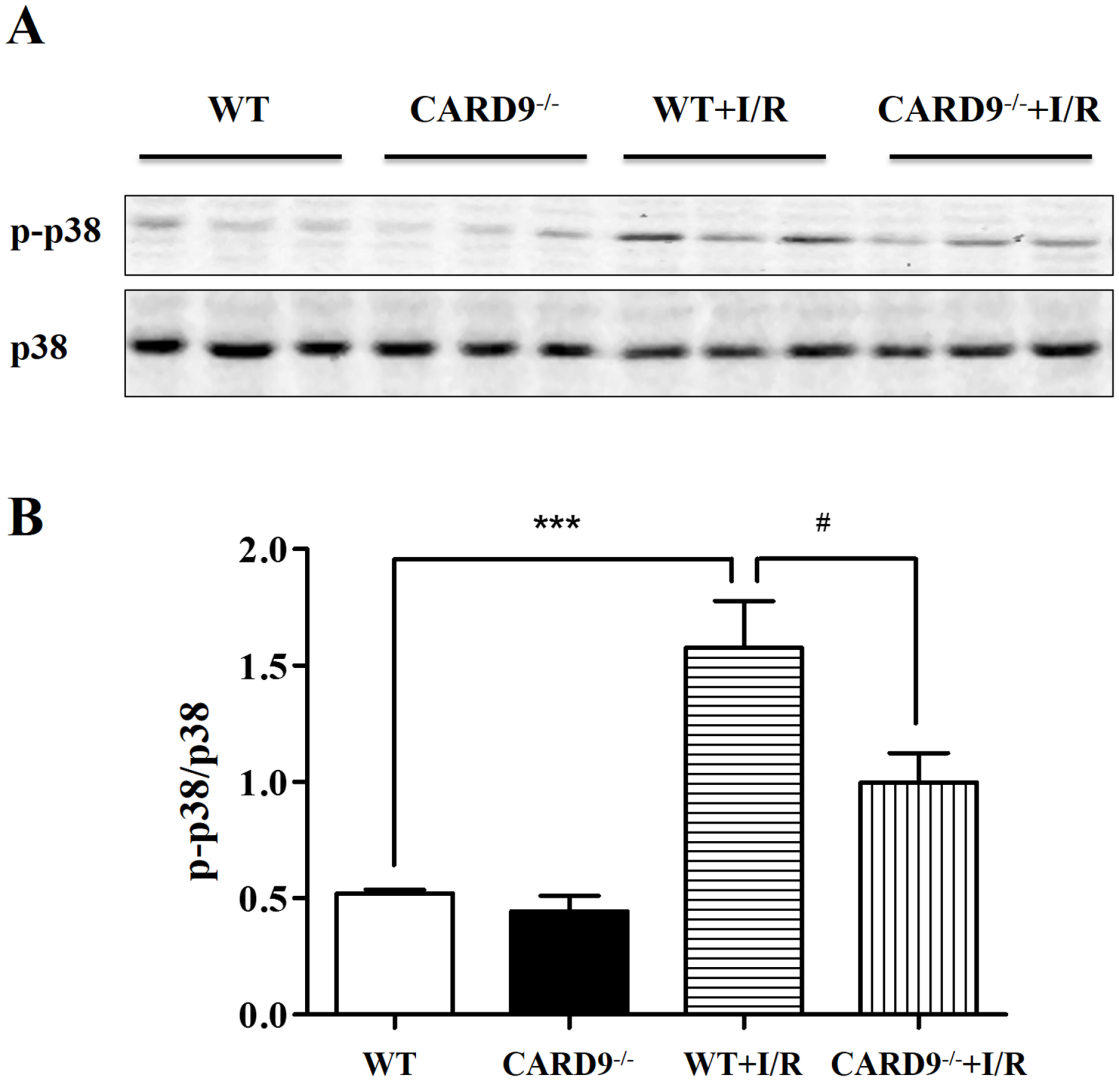
Western immunoblotting analyses of protein expressions in the heart. Following reperfusion, heart tissue from the risk area was harvested and homogenized. Western immunoblotting analyses were performed to measure the expression levels of phospho-p38 MAPK and p38 MAPK from WT and CARD9^-/-^ sham controls, WT+I/R, and CARD9^-/-^+I/R mice. A, Representative immunoblots of p-p38 MAPK and p38 MAPK; B, statistical analyses of the ratio of p-p38 MAPK/p38 MAPK. The phosphorylation level of p-38 MAPK was dramatically increased following I/R injury compared to sham controls. However, the ratio was significantly attenuated in CARD9^-/-^+I/R mouse heart compared to WT+I/R mouse heart. Mean ± SEM, n = 6/group, ***p < 0.001 vs. sham control; ^#^*p* < 0.05 vs. WT.

Activation of p38 MAPK has been reported to contribute to a number of pathological conditions [33–35]. In addition, p38 MAPK has also been suggested as one of the down-stream transcriptional factors of the CARD9 signaling complex in immune cells [18–20]. To determine if CARD9 knockout protects myocardial I/R injury through regulation of p38 MAPK signaling, Western immunoblotting analyses were performed on myocardial p38 MAPK phosphorylation levels. The ratio of phospho-p38 MAPK over p38 MAPK (p-p38/p38) was measured in heart tissue from WT and CARD9^-/-^ sham controls, WT+I/R, and CARD9^-/-^+I/R mice. As shown in Fig 4, I/R induced an approximately 3-fold increase in the ratio of p-p38/p38, while CARD9 knockout significantly reduced this increase indicating a potential connection between CARD9 and myocardial p38 MAPK signaling.

### CARD9 deficiency attenuated cytokine production in the heart and serum following I/R injury

**Fig 5:**
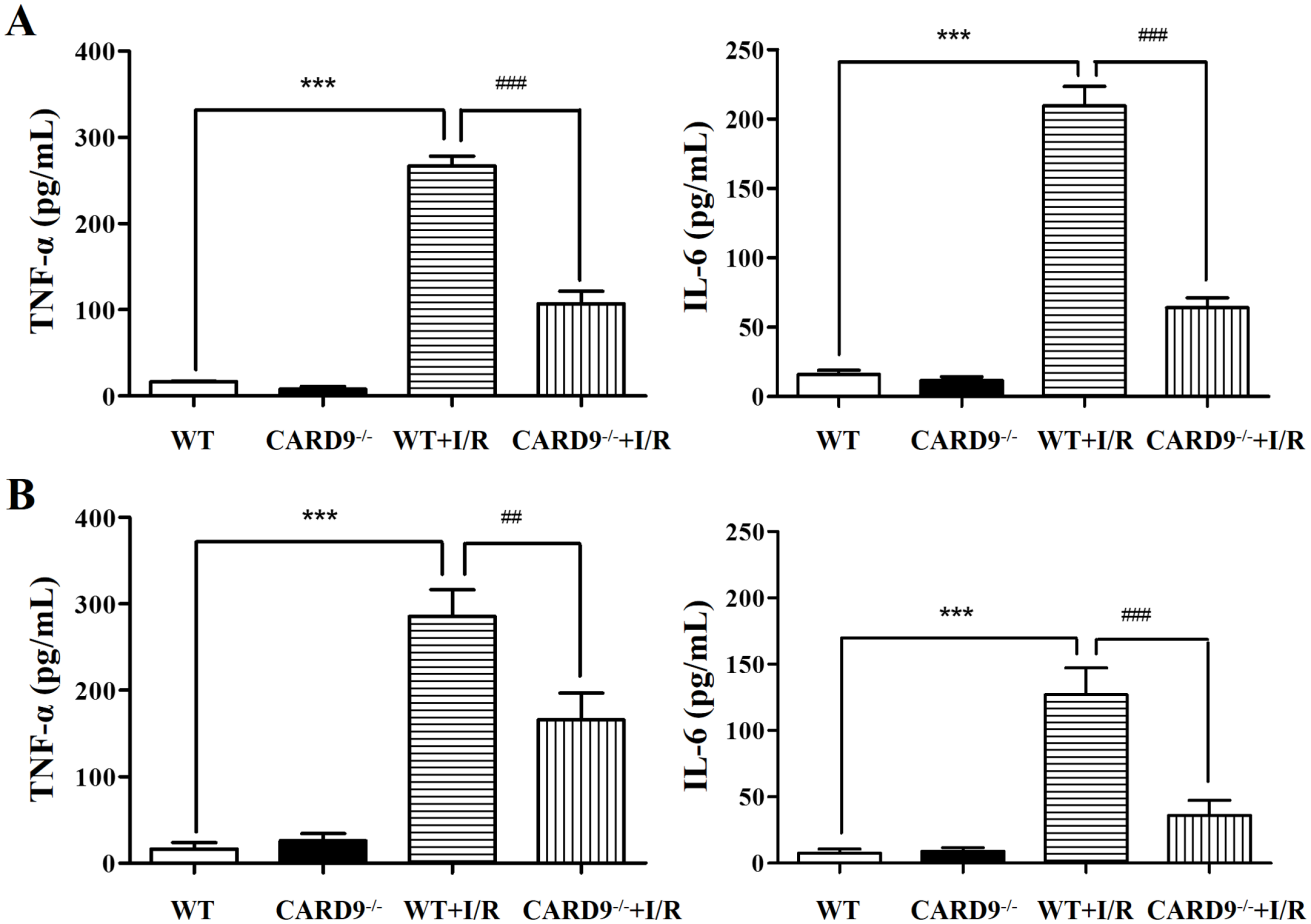
Measurement of cytokine production in heart tissue and serum in response to I/R injury. Following I/R injury, heart tissue and blood were harvested from WT and CARD9^-/-^ sham controls, WT+I/R and CARD9^-/-^+I/R mice. TNF-α and IL-6 were measured using commercial ELISA kits. A, Concentrations of TNF-α and IL-6 in heart tissue; B, concentrations of TNF-α and IL-6 in serum. I/R injury significantly increased the production of TNF-α and IL-6 compared to sham controls. CARD9 knockout significantly attenuated the levels of these two cytokines in heart tissue and serum following I/R injury. Mean ± SEM, n = 4/group, ***p < 0.001 vs. sham controls; ^##^*p* < 0.01, ^###^*p* < 0.001 vs. WT.

As I/R injury is associated with heightened cytokine production, to determine if CARD9 knockout affects the pathological activation of cytokines including TNF-α and IL-6, heart tissue and serum were harvested from both WT and CARD9^-/-^ sham controls, as well as from WT+I/R and CARD9^-/-^+I/R mice. TNF-α and IL-6 were measured using commercial ELISA kits as described in the Methods section. As shown in Fig 5, I/R injury significantly increased the production of TNF-α and IL-6 compared to the sham controls. Additionally, CARD9 knockout significantly attenuated the levels of these cytokines in the heart and serum.

### CARD9 deficiency attenuated muramyl dipeptide (MDP)-induced cytokine/chemokine production from co-cultured neutrophils

**Fig 6:**
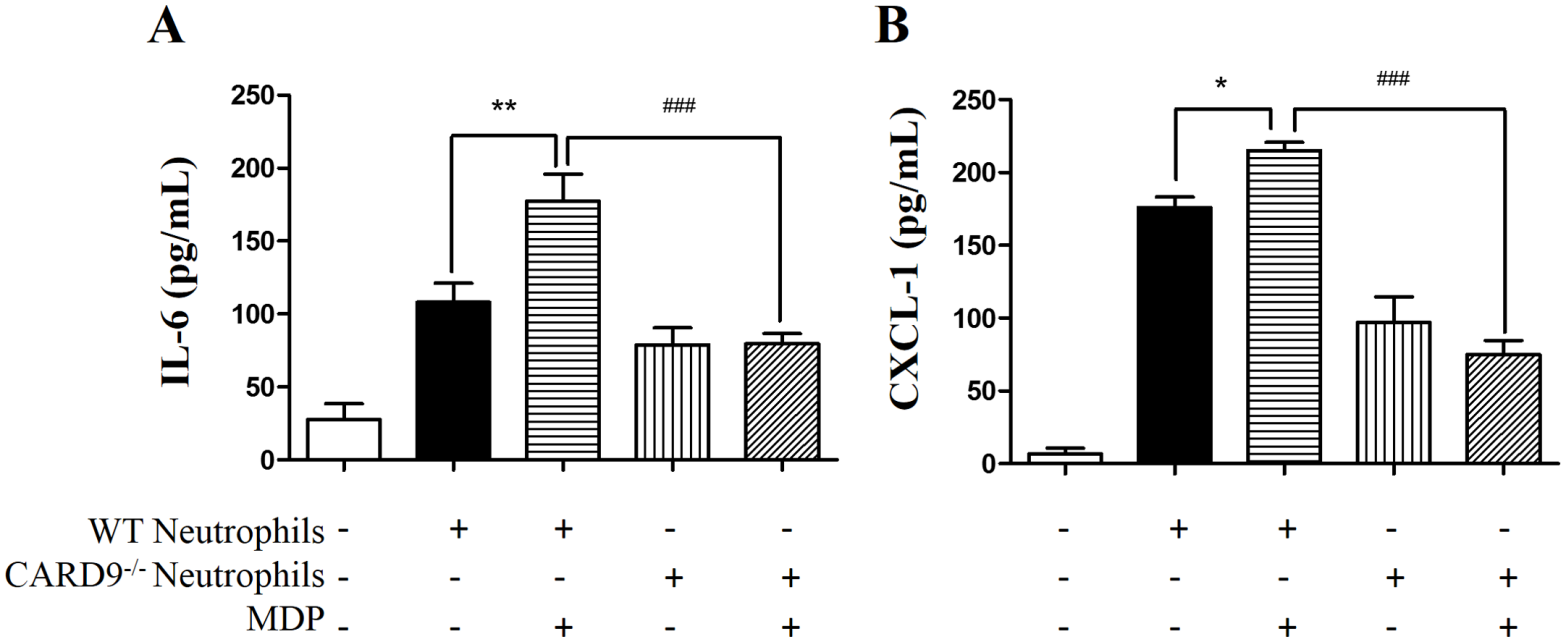
Measurement of cytokine/chemokine production in response to neutrophil activation in vitro. Neutrophils were isolated from WT and CARD9^-/-^ mice and co-cultured with H9C2 cells. MDP was used as an agonist to activate the CARD9 signaling. Cytokine/chemokine production was measured with commercial ELISA kits. The concentrations of IL-6 (A) and CXCL-1 (B) in the supernatant after 24 h co-culturing of the neutrophils with the H9C2 cells. Mean ± SEM, n = 6/group, *p < 0.05, **p < 0.01 vs. non-MDP treatment in the WT neutrophils; ^###^*p* < 0.001 vs. WT.

As CARD9 is specifically expressed in immune cells including neutrophils [18], we postulated that both neutrophil infiltration and cytokine/chemokine production contribute to I/R injury seen in cardiomyocytes. To determine whether or not CARD9 activation plays a role in the induction of cytokines/chemokines including IL-6 and CXCL-1, neutrophils were isolated, co-cultured with H9C2 myoblasts, and treated with MDP, a specific agonist of the CARD9 signaling complex as described in the Methods section [17]. As shown in Fig 6, when WT neutrophils were co-cultured and treated with MDP, the concentrations of IL-6 and CXCL-1 released in the supernatant were significantly elevated compared to the non-treated groups. Interestingly, when neutrophils from CARD9^-/-^ mice were co-cultured and treated with MDP, there was no significant alteration in the levels of IL-6 and CXCL-1 in the supernatant. These results indicated that MDP-elicited cytokine/chemokine production was dependent on the presence of CARD9 protein and associated signaling cascades.

## Discussion

As ischemic heart disease still leads all causes of morbidity and mortality, identifying risk factors and understanding underlying signaling pathways is critical for developing new pharmacological interventions. Following acute-phase ischemia and reperfusion, neutrophil infiltration into the infarcted myocardium adversely contributes to injury. However, the mechanisms underlying neutrophil infiltration and the subsequent chemokine/cytokine production are still not completely understood. As a central regulatory scaffold protein, CARD9 plays an essential role in the innate and adaptive immune responses. However, whether CARD9 participates in acute phase inflammation following I/R has not been investigated. The current study was designed to elucidate the potential role of CARD9 in contributing to myocardial I/R injury.

There are several interesting findings from the current study that are summarized in the following. First, CARD9 knockout protected the heart from ischemia and reperfusion injury as indicated by significantly reduced infarct size from CARD9^-/-^ mouse hearts compared to those from WT control mice (Fig 1). CARD9 is primarily expressed in immune cells including neutrophils and is required for responses to external infection [18–20]. Our results suggested that the same defense mechanism that affords protection against foreign insults is recruited in response to non-pathogenic (sterile) I/R injury and inflammation. Recent publications also pointed to similar defense mechanisms against sterile injury through DAMPs [5, 36, 37]. Our study identifies a potentially important protein mediator of internally-elicited inflammation in addition to the reported toll-like receptor (TLR)-associated pattern recognition receptor (PRR) signaling mechanisms.

Secondly, following I/R injury, neutrophil infiltration into the risk area was attenuated in the CARD9^-/-^ mouse heart. This is important given the fact that neutrophil infiltration is largely detrimental to the injured tissue [9, 13, 32, 38]. Given reduced neutrophil infiltration, one would expect less acute damage. Our study indicated that neutrophil infiltration following myocardial infarction is at least partially regulated by CARD9. Further, CARD9 knockout significantly reduced chemokine production (MCP-1 and CXCL-1) in the heart and serum which is consistent with the observed reduction in neutrophil trafficking into the at risk myocardium.

Thirdly, CARD9 knockout significantly attenuated the phosphorylation level of p38 MAPK in the myocardium (Fig 4). Activation of p38 MAPK has been implicated in the exacerbation of myocardial I/R injury resulting in myocyte apoptosis [34, 35] and other risk factor-associated myocardial dysfunctions [33]. Our results are consistent with findings that suppressed p38 MAPK phosphorylation in CARD9^-/-^ mice is associated with protection against myocardial injury. Interestingly, p38 MAPK also serves as a major downstream transcriptional factor of the CARD9 signaling complex in innate and adaptive immune responses [18]. Our results suggest a new potential mechanistic link between CARD9 signaling in infiltrated neutrophils and p38 MAPK activation-induced myocardial injury. IL-6 is an early responder to injury of cardiomyocytes and TNF-α is a major cytokine produced downstream of CARD9-activated p38 MAPK. These cytokines were significantly up-regulated following I/R injury. The findings that CARD9 knockout significantly reduced production of TNF-α and IL-6 in the serum and heart tissue (Fig 5) suggests that CARD9 knockout-conferred protection is at least partly due to reduction of injurious cytokine production.

Finally, cell-culture experiments were performed to determine the direct interactive signaling between neutrophils and cardiomyocytes. As shown in Fig 6, stimulation of neutrophils with MDP significantly increased the concentrations of CXCL-1 and IL-6. As expected, CARD9 knockout completely abrogated the production of each. These results substantiate our *in vivo* observations of the CARD9 knockout-associated protection. As MDP is a specific intracellular agonist to a protein complex partner of CARD9, nucleotide-binding oligomerization domain-containing protein 2 (NOD2), our results also suggested that activation of CARD9 signaling is responsible for the up-regulated chemokine/cytokine production. This interpretation of our *in vitro* findings is supported by a recent paper that suggests CARD9 deficiency also impairs neutrophil function against fungi infection [39]. Consistent with our findings, NOD2 knockout was protective against myocardial infarction [40].

In conclusion, CARD9 knockout protected against myocardial I/R injury and reduced chemokine and cytokine production, neutrophil infiltration, and p38 MAPK phosphorylation. As CARD9 signaling is a requisite component of immune responses to pathogens, our results indicate a new signaling relationship between immune and sterile inflammatory responses to I/R thus providing a potential new target with the overall aim of myocardial I/R injury reduction. Further studies are warranted on the potential mechanistic link between apoptotic/necrotic signaling and the activation of CARD9 signaling.

## Supporting information

S1 File phospho-p38 MAPK immunoblot. Supporting file for Fig 4.
(1SC)

S2 File p38 MAPK immunoblot. Supporting file for Fig 4.
(1SC)
